# A Comparative Study of Involvement and Motivation among Casino Gamblers

**DOI:** 10.4306/pi.2009.6.3.141

**Published:** 2009-07-08

**Authors:** Choong-Ki Lee, BongKoo Lee, Bo Jason Bernhard, Tae Kyung Lee

**Affiliations:** 1 College of Hotel & Tourism, Kyunghee University, Seoul, Korea.; 2 Department of Tourism Management, Dong-Eui University, Busan, Korea.; 3 Department of Hotel Management and Sociology, University of Nevada, Las Vegas, NV, USA.; 4 Department of Addiction Psychiatry, Seoul National Hospital, An Affiliate of Ministry for Health, Welfare & Family Affairs, Seoul, Korea.

**Keywords:** Involvement, Motivation, Non-problem gambler, Some problem gambler, Probable pathological gambler

## Abstract

**Objective:**

The purpose of this paper is to investigate three different types of gamblers (which we label "non-problem", "some problem", and "probable pathological gamblers") to determine differences in involvement and motivation, as well as differences in demographic and behavioral variables.

**Methods:**

The analysis takes advantage of a unique opportunity to sample on-site at a major casino in South Korea, and the resulting purposive sample yielded 180 completed questionnaires in each of the three groups, for a total number of 540. Factor analysis, analysis of variance (ANOVA) and Duncan tests, and Chi-square tests are employed to analyze the data collected from the survey.

**Results:**

Findings from ANOVA tests indicate that involvement factors of importance/self-expression, pleasure/interest, and centrality derived from the factor analysis were significantly different among these three types of gamblers. The "probable pathological" and "some problem" gamblers were found to have similar degrees of involvement, and higher degrees of involvement than the non-problem gamblers. The tests also reveal that motivational factors of escape, socialization, winning, and exploring scenery were significantly different among these three types of gamblers. When looking at motivations to visit the casino, "probable pathological" gamblers were more likely to seek winning, the "some problem" group appeared to be more likely to seek escape, and the "non-problem" gamblers indicate that their motivations to visit centered around explorations of scenery and culture in the surrounding casino area.

**Conclusion:**

The tools for exploring motivations and involvements of gambling provide valuable and discerning information about the entire spectrum of gamblers.

## Introduction

Some gambling researchers have suggested that gambling behaviors can be considered on a continuum, ranging from gambling without any significant problems through severe levels of pathological gambling. Previously, researchers^1^,^2^ have claimed that gamblers can be classified into three groups: gamblers with no problems, gamblers with some problems, and probable pathological gamblers. The category of "gamblers with no problems" has also been called social gamblers^2^ or recreational gamblers.^3^,^4^

A number of studies have found that different types of gamblers tend to express different motivations to gamble. For example, researchers^3^ have differentiated between these three groups by employing the Recreational Experience Preference (REP) Scale, which examines 23 motives for gambling. The results of their study indicate that pathological gamblers ranked the importance of REP motives for gambling significantly higher than the group of gamblers with some problems ranked them. They also found that the group of "gamblers with some problems" ranked the importance of REP motives higher than gamblers with no problems. Another group of researchers^3^ examined gambling motivations among different groups of student gamblers. The results of their study indicate that significant motivational differences exist between recreational and pathological gamblers in this sample.

Other studies have examined general gambling motivations among sub-populations-without a focus on pathology. For instance, a separate student study^5^ explored motivations to gamble with a general sample of college students and identified the following as primary motivations: money, fun, socialization, excitement, passing time, winning, conformity, competition, risk-taking, interest, skill, escape, chasing, drinking, and challenge. Meanwhile, a study of elderly female gamblers reported that participants were motivated to gamble for reasons pertaining to entertainment, excitement, people watching, and escape from routine.^7^ A separate study^8^ built upon this research by suggesting that seniors who may feel a loss of control over their lives can not only regain a sense of control when they gamble, but can also achieve a type of escape from their physical and emotional constraints presented by their everyday lives.

In contrast to the growing literature on gambling motivations, the concept of involvement has not received much attention in the field of gambling studies. The concept has been generally defined as the personal meaning or affective attachment an individual has for an activity or a setting,^9^,^10^ and as such it has been regarded as having an enduring nature.^11^ Researchers have suggested that the concept incorporates at least four underlying factors: pleasure/interest, centrality to lifestyle, perceived importance, and self-expression.^9^,^12^-^14^ These researchers have come to recognize involvement as a potentially powerful explanatory variable-one that can help enhance our understanding of a variety of leisure activities. For instance, a study^12^ suggested that the concept of involvement could explain why people participate in different types of activities and use different types of facilities in a fitness club.

It has been further argued that the involvement concept serves as one of the most important factors in explaining why and how people develop interests and skill levels in recreational activities-and, hence, how they develop into a "specialist" in an activity.^9^ Specialists tend to display a high degree of commitment, activity-related knowledge, and focus to a degree that the activity becomes a central life interest.^15^ Earlier studies found that the concept of involvement is related to the typology of leisure participants, ranging from novice to specialist.^16^ Most importantly for our purposes is one researcher's claim^17^ that although in common usage the term "specialization" often possesses a positive connotation, sometimes it can take a negative turn. This researcher argued that there were costs to becoming serious specialists, especially as family members, friends, and others can frequently misunderstand the recreational lives of serious leisure participants. A darker side of specialization is also revealed when serious leisure seekers become "addicted" to the recreational activity-a notion worth exploring, in our view, when the activity of choice is gambling.

In the gambling literature, only a single study^18^ has investigated the relationship between involvement and gambling behavior. In this study, involvement was found to be composed of three dimensions: self-identity/self-expression, pleasure/interest/importance, and centrality-contrary to the usually suggested four dimensions (self-expression, pleasure/interest or enjoyment, centrality, and importance). This study's results also indicated that pleasure (enjoyment) and centrality were the two most important dimensions of involvement for gamblers. Finally, this study suggested that future research should further engage these involvement dimensions when exploring the nuances of gambling behavior.

In this paper we will attempt to answer this call by further exploring dimensions of involvement for gamblers in three gambling groups. Overall, this paper seeks to achieve three objectives: 1) to identify the underlying dimensions of involvement and motivation in a sample of Korean casino gamblers, using a factor analysis; 2) to explore any differences in involvement and motivation among three types of gamblers (non-problem, some problem, and probable pathological gamblers); and 3) to examine any differences among these three types of gamblers with respect to demographic and behavioral variables.

## Methods

### Participants and procedure

Because we wanted to be able to compare across the three gambling groups outlined by researchers,^1^-^3^ a purposive sampling method was deemed most appropriate. This method yielded 180 completed questionnaires for "non-problem gamblers", 180 completed questionnaires for the "some problem gamblers" group, and 180 completed questionnaires for the group of "probable pathological gamblers". To ensure that a more representative sample of South Korean gamblers was achieved, the survey was conducted with actual casino gamblers on both weekdays and weekends in the middle of June 2002. Questionnaires were administered at a temporary booth in the casino, allowing for unusual access to gamblers during actual gambling visits-a situation which should enhance recall accuracy. Field researchers approached casino gamblers, outlined the purpose of the research project, and invited them to participate in the survey. After consenting, a self-administered questionnaire was presented to each respondent. The questionnaires were then completed in the presence of the field researchers, allowing for rigorous monitoring of the data collection process.

### Measures

The involvement items employed in our research instrument were based upon similar items in previous research.^9^,^11^,^19^-^21^ Using a 5-point Likert scale, respondents were asked to indicate their levels of agreement with statements based upon these items (1=strongly disagree, 3=neutral, 5=strongly agree). All items were pre-tested with casino visitors, and participants in this pre-test were asked to evaluate the appropriateness of the measuring instruments. After the pre-test, questions that were poorly understood were reworded for clarity, and a final list was generated of 15 involvement items associated with casino gambling.

Similarly, a preliminary list of motivation items was generated from a review of related literature on gambling motivations.^5^-^7^,^22^,^23^ In addition to these previously-explored issues, other "new" items explored the matters of whether subjects were motivated to visit the casino by features outside of the casino-in this case, both the mountain scenery surrounding the area and the cultural and historical attractions nearby. These kinds of oft-neglected motivations are perhaps increasingly important as a variety of gambling settings attempt to synthesize gambling activities with other nearby non-gambling activities. In sum, not all visitors to casino spaces were there just for the gambling, and we wished to explore these motivations in our instrument. As with the involvement items, these items were pre-tested with casino visitors, and respondents were asked to evaluate the appropriateness of the measuring instruments. After the pre-test, questions that were poorly understood were reworded for clarity. These procedures yielded a list of 24 motivational items associated with casino gambling.

The problem gambling scale used in this project was the Gamblers Anonymous Twenty Questions (GA20). This instrument has been widely employed in a variety of research and clinical settings.^1^,^2^,^24^ Originally developed by Gamblers Anonymous members to identify the existence and severity of a gambling problem,^25^ it has since been used in research settings as well. This instrument was developed by those with gambling problems, giving it an immediate face validity.^1^ In a psychometric study seeking to test the reliability and validity of the measure,^24^ researchers suggested that this non-medical background is important, and concluded by hailing its "extraordinary performance" (p.12) as a research and clinical instrument. This study also suggested that its convergent validity is high when measured against the widely used South Oaks Gambling Screen, and a factor analysis revealed that its construct validity is also high. In a final measure of validity, this study suggests that the instrument is highly capable of distinguishing problem gamblers, giving it high discriminatory validity, and that it also demonstrates high diagnostic efficacy (98.9%). Because the GA20 emphasizes common, everyday problems associated with gambling, and because it avoids medical constructs such as tolerance, we believed that this instrument was appropriate for translation and administration in South Korea. Notably, the only study that evaluates the reliability and validity of the instrument^24^ not only supported its use-it also involved a translation into a different language and culture.

As with previous research,^1^,^3^,^4^ our study classifies gamblers into three groups by assessing total scores of the GA 20: 1) a score of 0 translates to "non-problems" with gambling; 2) a score between 1 and 6 is indicative of "some problems" with gambling; and 3) a score higher than 7 categorizes the respondent as a "probable pathological gambler".

## Results

### Factor analysis of gambling involvement

Factor analysis is a statistical method used to derive underlying dimensions that explain data in a much smaller number of concepts than the original individual variables.^26^ Common criteria in extracting these factors^27^ are: 1) eigenvalues representing the amount of variance explained by a factor (these should greater than 1); 2) factor loadings accounting for the correlation between the original variables and each factor (these should be greater than 0.4); and 3) reliability coefficients to check the internal consistency of items with each factor (these should be above 0.6).

In our case, fifteen items of gambling involvement were factor analyzed using the principal component method and varimax rotation procedure to uncover underlying meaningful dimensions. Three items with factor loading lower than 0.4 were removed and the remaining twelve items were factor analyzed again. The final factor analysis resulted in three underlying dimensions of gaming involvement. As shown in Table 1, all three factors had eigenvalues greater than 1, reliability coefficients ranging from 0.65 to 0.87, and accounted for 63.1% of the total variance. Hence, these factors appeared to exceed the minimum appropriate standards.^28^

The first factor, labeled "importance and self-expression", explained 40.8% of the total variance, with a reliability coefficient of 0.87 (Table 1). The relatively large proportion of the total variance for the factor leads us to conclude that "importance and self-expression" is of central importance to our sample of gamblers. The second factor was labeled "pleasure and interest", and accounted for 12.2% of the variance with a reliability coefficient of 0.73. The final factor was labeled "centrality", and explained 10.0% of the variance with a reliability coefficient of 0.65.

### Differences in involvement among three types of gamblers

In Table 2, we shift our attention to examine differences in involvement among the sub-samples of non-problem, some problem, and probable pathological gamblers. In examining the involvement factors, analysis of variance (ANOVA) tests reveal that the factor of "importance and self-expression" was significantly different at p<0.01 among these three subgroups. Next, Duncan's multiple-range tests were performed to further examine differences in involvement among the three types of gambling segments. The results of these tests indicate that the three types of gamblers were significantly different with respect to the factor of importance and self-expression. Mean values also indicate that gamblers with severest problems scored highest on this factor: probable pathological gamblers had the largest value for this factor, some problem gamblers had the second largest value, and the gamblers with no problems had the lowest value.

The ANOVA tests also indicate that the factor of "pleasure and interest" was significantly different among three types of gambling segments at p<0.01. Duncan's multiple-range tests reveal that the gamblers with no problems were statistically different from the some problem and probable pathological gambler groups, whereas no significant difference was found between the some problem and probable pathological gamblers. Put another way, the gamblers with problems (both "some problem" and "probable pathological" groups) in our sample scored higher on the "pleasure and interest" factor than gamblers without problems.

Finally, ANOVA tests also reveal that the centrality factor was significantly different among these three groups of gamblers at p<0.01. Duncan's multiple-range tests showed that the non-problem group was statistically different from the groups of some problem and probable pathological gamblers, whereas no significant difference was found between the some problem and probable pathological gamblers. Once again, this means that the "some problem" and "probable pathological" gamblers are similar on the centrality factor, but they are both different from the gamblers with no problems (at least with respect to this factor).

### Factor analysis of motivation

Next we turn our attention to gambling motivation. Twenty four motivational items were factor analyzed using the principal component method and varimax rotation procedure, a technique that enables researchers to isolate meaningful underlying dimensions of motivation. Five items with factor loading lower than 0.4 were removed, and the remaining nineteen items were factor analyzed again. The final factor analysis resulted in four underlying dimensions of motivations. As shown in Table 3, all four factors had eigenvalues greater than 1. These factors had reliability coefficients ranging from 0.69 to 0.89, and accounted for 63.0% of the total variance. Thus, these factors appeared to exceed the appropriate minimum standards.

The first factor was labeled "escape", and explained 33.9% of the total variance with a reliability coefficient of 0.89. The second factor was labeled "socialization", and accounted for 14.6% of the variance with a reliability coefficient of 0.82. The third factor was labeled "winning", and explained 8.9% of the variance with a reliability coefficient of 0.69. The final factor was labeled "scenery and culture", and accounts for 5.6% of the variance with a reliability coefficient of 0.70. Based upon this latter finding, it would seem that our belief that non-gambling motivations are important for certain visitors is supported.

### Differences in casino motivation among three types of gamblers

Next, we examine the three sub-groups of gamblers and their motivations. Table 4 presents differences in motivations for casino gambling among gamblers with no problems, gamblers who have some problems, and gamblers who qualify as probable pathological gamblers.

The ANOVA tests reveal that the motivational factor of escape was significantly different at p<0.01 among these groups. Once again, Duncan's multiple-range tests were also performed to further examine differences in casino motivations among the three types of gamblers. The results of these tests yielded some interesting and non-linear results, in that gamblers with no problems and probable pathological gamblers were similar on the factor of escape, but they were different from the "some problems" group with respect to this factor. However, mean values of this factor show that the some problem gamblers had the largest value, the probable pathological gamblers had the second largest value, and the gamblers with no problems had the lowest value-which seems to indicate that those with problems tend to be motivated more by escape.

The ANOVA tests also indicate that the socialization motivational factor was significantly different at p<0.01 among these three groups. Duncan's multiple-range tests reveal that with the socialization factor, the gamblers with no problems were statistically different from the some problem and probable pathological gamblers, and no significant differences were found between the latter two groups. This implies that the gamblers without problems are different from the some problem and probable pathological gamblers, but the last two gambling groups are similar-at least in terms of their expressed motivations to gamble as it is measured on this socialization factor.

Turning our attention to the next analysis, ANOVA tests also reveal that the "winning" motivational factor was also significantly different at p<0.01 among the three groups of gamblers. Duncan's multiple-range tests reveal that the three groups were statistically different from each other with respect to the winning factor. Mean values indicate that motivations for winning were strongest among probable pathological gamblers, followed by the some problem and non-problem groups, respectively. Put another way, those who were motivated by winning tended to be among the most problematic in our sample.

When we turn our attention to non-gambling motivational factors, we again find significant differences. The ANOVA tests on these items reveal that the factor of "scenery and culture" was significantly different at p<0.01 among three types of gambling segments. Duncan's multiple-range tests indicate that gamblers with no problems were statistically different from probable pathological gamblers, whereas no significant differences were found between non-problem and some problem gamblers.

### Differences in demographic characteristics among the three types of gamblers

In order to further identify the profile of the three groups of gamblers examined here, each segment was cross-tabulated with socio-economic variables and selected behavioral variables. As shown in Table 5, the results of the Chi-squire tests indicate that there were statistically significant differences among the three gambling groups: for the "some problem" and "probable pathological" gambling groups, gambling was the primary purpose of their visit to casino. On the other hand, gambling was not the major purpose of the casino visit with the "non-problem" group, who tended to cite non-gambling factors in articulating their decision to come to the casino. Perhaps predictably, length of stay for the gamblers with no problems appeared to be shorter relative to the other two gambling segments. In addition, gamblers with no problems tended to visit the casino less frequently than the some problem and probable pathological gamblers did.

The probable pathological gamblers were more likely to be alone when they gambled, whereas the gamblers with no problems tended to be accompanied by family, and the some problem gamblers tended to be accompanied by friends and relatives. The probable pathological gamblers were more likely to prefer baccarat and blackjack, whereas the "non-problem" gamblers were more likely to prefer slot machines. Meanwhile, the intermediate some problem gambling group tended to prefer both slot machines and blackjack.

Demographically, the some problem and probable pathological gamblers were characterized by a relatively high number of males, whereas the gamblers with no problems were characterized by a relatively equal distribution of males and females.

### Differences in preference of recreational activities among the three types of gamblers

In addition to measurements of gaming involvement and motivations, respondents were asked about their other recreational preferences during their visit to the resort. To this end, ANOVA tests were conducted to observe any differences in preferences among three types of gamblers. As shown in Table 6, the results of ANOVA and Duncan's multiple-range tests indicate that for outdoor recreational facilities, such as the golf course, ski lift, and theme park, the "non-problem" gamblers were similar to the "some problem" gamblers, but the first two segments were different from the "probable pathological" gamblers. Mean values show that the "non-problem" and "some problem" gamblers were more likely to prefer outdoor recreational activities than the "probable pathological" gamblers were.

On the other hand, for indoor activities the results looked a bit different: the some problem gamblers appeared to be similar to the probable pathological gamblers, but they were different from the gamblers with no problems. Mean values also show that the "probable pathological" gamblers and "some problem" gamblers were more likely to prefer the indoor activities of 'indoor sports facilities' and 'cinema' than those without problems. In sum, the evidence here suggests that gamblers with problems appear to prefer indoor activities, while those without problems prefer outdoor activities.

## Discussion

This study aimed to investigate differences in involvement and motivations among three types of gambling groups: gamblers with no problems, gamblers with some problems, and probable pathological gamblers.

The findings on involvement appeared to be conceptually clear and similar to findings in previous research.^29^ However, one difference emerged: in our study, the factor of importance and self-expression emerged within the same dimension, and the pleasure factor emerged as a separate dimension. The findings on motivation were also similar to previous research,^27^ except for the addition of one significant factor: "scenery and culture".

These results also show that the "some problem" and "probable pathological" gamblers had higher levels of involvement in casino gambling than the non-problem gamblers, indicating that this construct has considerable analytical value. Involvement measurements-long neglected in the gambling field, but important in the leisure field-may well help differentiate those with gambling problems from those without any gambling problems. Put another way, there does indeed appear to be a "dark side" to intense levels of involvement.

When it comes to motivations, the "probable pathological" gamblers appeared to be similar to the "some problem" gamblers in seeking socialization and escape, indicating that these kinds of motivations are important to those who develop problems with gambling. This finding implies that the escape item may contribute significantly to explaining problematic gambling, a finding that is consistent with previous speculations on dissociative gambling.^30^

Overall, mean values show that the "some problem" gamblers were most likely to be motivated by escape, the "probable pathological" gamblers were most likely to be motivated by winning, and the gamblers with no problems were most likely to be motivated by the local mountain scenery and culture. This latter point is perhaps most telling with regard to the increasingly prominent development of non-gambling amenities adjacent to casino gambling locations. Specifically, it would seem that those who exhibit problems with their gambling are interested primarily in the gambling itself, while the non-gambling amenities motivate those who do not display any problematic behaviors.

In terms of preference for recreational activities outside of the gambling environment, the finding seems to indicate that "probable pathological" gamblers may well prefer the indoors to the outdoors, while those without problems seem to prefer to "head outside"-to activities that extend beyond the casino walls. Once again, "healthier" gamblers appear to be those with a variety of recreational interests outside of the casino, providing support for a general belief that is embodied in gaming jurisdictions like Singapore and Las Vegas.

Overall, then, it seems that the probable pathological gamblers tend to be uninterested in a broader range of recreational activities outside of casino gambling-while those who do not qualify as having problems engage in a broader array of recreations in and around the casino environment. This is a telling finding, as it seems to indicate that new gaming jurisdictions that emphasize this "total recreation destination" approach (such as Singapore), as well as existing jurisdictions that are expanding their offerings to embrace this kind of approach (such as Las Vegas) are on the right track-at least from a social impact perspective.

In conclusion, it seems that when applying the (occasionally neglected) "tools" of leisure research to the specialized and burgeoning field of gambling research, these tools do indeed provide valuable and discerning information about the entire spectrum of gamblers. In the future, leisure researchers and gambling researchers would do well to further explore parallel interests and theoretical constructs, with hopes of developing a coherent and consistent understanding of those who choose to gamble with their leisure time. As gambling becomes an increasingly central leisure-time pursuit in a growing number of international jurisdictions, it would seem that gambling researchers' and leisure researchers' futures are inextricably linked.

## Figures and Tables

**TABLE 1:**
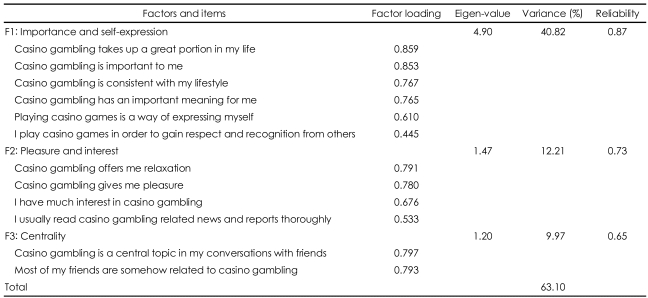
Results of factor analysis for gambling involvement

**TABLE 2:**
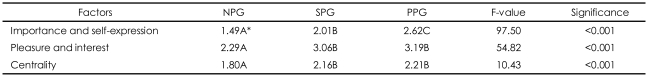
Differences in involvement among three types of gamblers using ANOVA

Measured with 5 point Likert-type scale: 1=strongly disagree, 3=neutral, 5=strongly agree. ^*^Duncan's multiple-range tests: means with the same letter are not significantly different from at 5% level. NPG: non-problem gamblers, SPG: some problem gamblers, PPG: probable pathological gamblers, ANOVA: analysis of variance

**TABLE 3:**
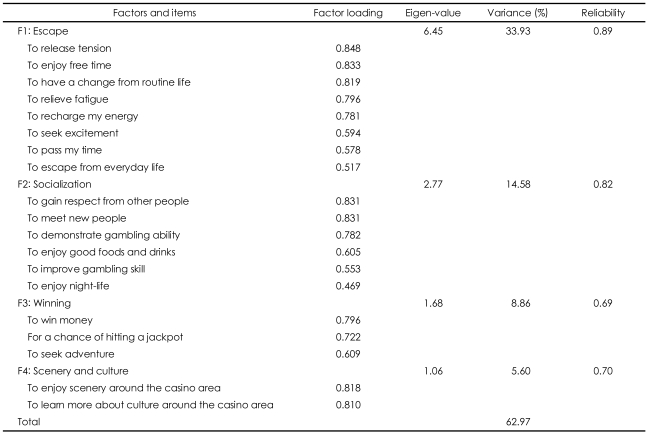
Results of factor analysis for gambling motivation

**TABLE 4:**
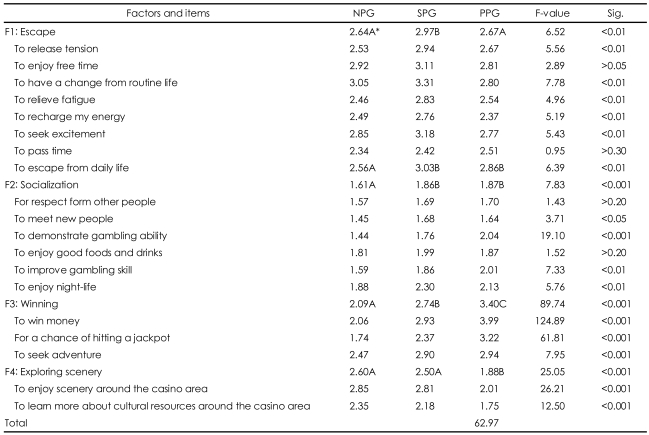
Differences in motivation among three types of gamblers using ANOVA

Measured with 5 point Likert-type scale: 1=strongly disagree, 3=neutral, 5=strongly agree. ^*^Duncan's multiple-range tests: means with the same letter are not significantly different from at 5% level. NPG: non-problem gamblers, SPG: some problem gamblers, PPG: probable pathological gamblers, ANOVA: analysis of variance

**TABLE 5:**
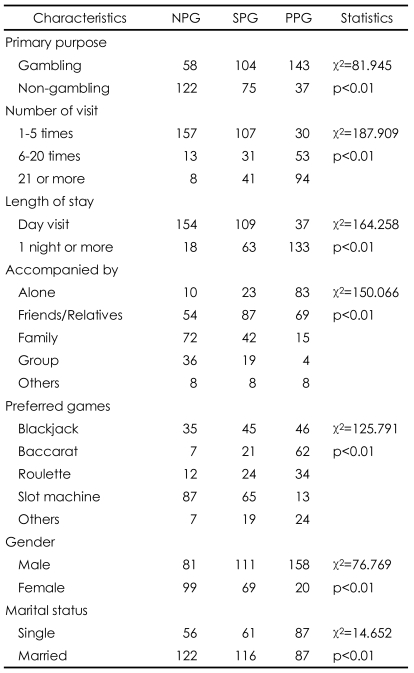
Differences in characteristics among three types of gamblers

NPG: non-problem gamblers, SPG: some problem gamblers, PPG: probable pathological gamblers

**TABLE 6:**
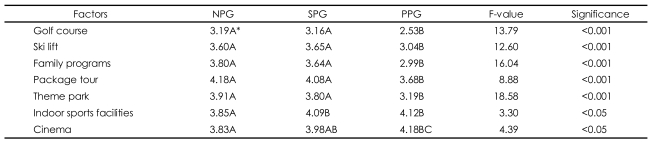
Differences in reference of recreational activities among three types of gamblers using ANOVA

Measured with 5 point Likert-type scale: 1=strongly disagree, 3=neutral, 5=strongly agree. ^*^Duncan's multiple-range tests: means with the same letter are not significantly different from at 5% level. NPG: non-problem gamblers, SPG: some problem gamblers, PPG: probable pathological gamblers, ANOVA: analysis of variance
